# ASL MRI informs blood flow to chronic stroke lesions in patients with aphasia

**DOI:** 10.3389/fphys.2023.1240992

**Published:** 2023-07-20

**Authors:** Lisa C. Krishnamurthy, Clara Glassman, Joo H. Han, Serena E. Song, Chanse Denmon, Maryanne Weatherill, Amy D. Rodriguez, Bruce A. Crosson, Venkatagiri Krishnamurthy

**Affiliations:** ^1^ Center for Visual and Neurocognitive Rehabilitation, Atlanta VA Health Care System, Decatur, GA, United States; ^2^ Joint GSU, Georgia Tech, and Emory Center for Translational Research in Neuroimaging and Data Science (TReNDS), Atlanta, GA, United States; ^3^ Department of Physics and Astronomy, Georgia State University, Atlanta, GA, United States; ^4^ Department of Radiology and Imaging Sciences, Emory University, Atlanta, GA, United States; ^5^ Department of Neurology, Emory University, Atlanta, GA, United States; ^6^ Division of Geriatrics and Gerontology, Department of Medicine, Emory University, Atlanta, GA, United States; ^7^ Department of Veterans Affairs (VA) Health Care System, Decatur, GA, United States

**Keywords:** chronic stroke lesion, cerebral blood flow, TIGR MRI, brain-behavior maps, stroke rehabilitation targets

## Abstract

**Introduction:** Response to post-stroke aphasia language rehabilitation is difficult to anticipate, mainly because few predictors can help identify optimal, individualized treatment options. Imaging techniques, such as Voxel-based Lesion Symptom Mapping have been useful in linking specific brain areas to language behavior; however, further development is required to optimize the use of structural and physiological information in guiding individualized treatment for persons with aphasia (PWA). In this study, we will determine if cerebral blood flow (CBF) mapped in patients with chronic strokes can be further used to understand stroke-related factors and behavior.

**Methods:** We collected perfusion MRI data using pseudo-Continuous Arterial Spin Labeling (pCASL) using a single post-labeling delay of 2,200 ms in 14 chronic PWA, along with high-resolution structural MRI to compute maps of tissue damage using Tissue Integrity Gradation via T2w T1w Ratio (TIGR). To quantify the CBF in chronic stroke lesions, we tested at what point spatial smoothing should be applied in the ASL analysis pipeline. We then related CBF to tissue damage, time since stroke, age, sex, and their respective cross-terms to further understand the variability in lesion CBF. Finally, we assessed the feasibility of computing multivariate brain-behavior maps using CBF and compared them to brain-behavior maps extracted with TIGR MRI.

**Results:** We found that the CBF in chronic stroke lesions is significantly reduced compared to its homologue grey and white matter regions. However, a reliable CBF signal (although smaller than expected) was detected to reveal a negative relationship between CBF and increasing tissue damage. Further, the relationship between the lesion CBF and age, sex, time since stroke, and tissue damage and cross-terms suggested an aging-by-disease interaction. This relationship was strongest when smoothing was applied in the template space. Finally, we show that whole-brain CBF relates to domain-general visuospatial functioning in PWA. The CBF-based brain-behavior maps provide unique and complementary information to structural (lesion-based) brain-behavior maps.

**Discussion:** Therefore, CBF can be detected in chronic stroke lesions using a standard pCASL MRI acquisition and is informative at the whole-brain level in identifying stroke rehabilitation targets in PWAs due to its relationship with demographic factors, stroke-related factors, and behavior.

## Introduction

The most common cause of acquired language impairment (also known as aphasia) is stroke. Aphasia presents in up to 40% of people who experience acute strokes and persists in approximately 61% of individuals after 1 year (Pedersen et al., 2004). Unfortunately, response to post-stroke aphasia rehabilitation is variable, and identifying the best treatment options for a specific patient has been difficult. Researchers are increasingly using structural and functional imaging to inform and augment treatment development, which heralds improved language outcomes (Meinzer et al., 2011; Crosson et al., 2017; Crosson et al., 2019). However, using imaging data to inform clinical decisions is still in its infancy. A review of imaging studies in aphasia reveals that a diverse range of lesion locations and subsequent brain physiology changes hamper efforts to select appropriate interventions to optimize treatment outcomes (Charidimou et al., 2014). However, there is evidence that mapping each patient’s unique brain anatomy (structural imaging) and physiology (cerebral blood flow imaging) to their language deficits (Fridriksson, 2010) could result in more individualized intervention.

Several imaging methodologies can provide associations between lesion location and language behavior, including Voxel-based Lesion Symptom Mapping (VLSM), which has been popular in stroke research since Bates et al. (2003) introduced the method in 2003. The technique acquires detailed images of the patient’s brain using high-resolution T1-weighted (T1w) anatomical Magnetic Resonance Imaging (MRI) scans. An experienced neuroimager then can manually demarcate the lesion. The area of the lesion is transformed into a subject-specific binary mask and entered into a t-test analysis to define which lesion location corresponds to specific language deficits. VLSM is an elegant algorithm that defines structure-behavior associations and implicitly takes advantage of the heterogeneity of language behavior and lesion location. Using functional and structural imaging to study persons with aphasia (PWA) facilitates linking a specific brain area to specific language behaviors and deficits. VLSM (Bates et al., 2003), is a simple yet elegant method used to define structure-behavior associations. However, the technique has several limitations in that it depends on binary “all-or-nothing” lesion masks to define areas of structural compromise and does not offer a comprehensive picture of brain health.

In 2005, Tyler et al. (2005) advanced VLSM by discarding the binary lesion masking step by correlating the continuous T1w image signal intensity with continuous language measures. According to Tyler et al. (2005), judging whether cortical tissue is intact or damaged with an “all-or-none distinction fails to capture a much larger range of potentially informative gradations in the degree of structural damage.” This is a reasonable assessment, as the damage from stroke is not limited to the Cerebral Spinal Fluid (CSF) filled cavitation, and includes regions of gliosis and Wallerian degeneration, all with varying impact on behavior. It was demonstrated that T1w image signal intensity has high correlations with word processing abilities using a lexical decision task. Though the small number of subjects (*n* = 19) could have led to issues with statistical power (Kimberg et al., 2007), this study established the feasibility and utility of correlating the MRI signal intensity with language behavior. Despite this important step forward, the methodology of using the T1w signal has some limitations in the MRI signal normalization process, as it is dependent on highly variable anatomical attributes across stroke survivors. To address the methodological issues from Tyler et al. (2005), we developed Tissue Integrity Gradation via T2w T1w Ratio (TIGR) MRI (Krishnamurthy et al., 2021) by using a ratio of T2w and T1w signals and by normalizing the signals using bounds determined by the grey matter and cerebrospinal fluid signal intensities of intact regions. Therefore, regardless of atrophy, headsize, or coil loading characteristics, the normalization procedure in TIGR is comparable across all participants within a cohort and does not require a control sample of intact brains to perform the analysis.

Although the predominant line of thinking by clinicians and scientists alike is that everything within the stroke lesion is necrotic, we have evidence that this may not be the case. Using Tissue Integrity Gradation via T2w T1w Ratio (TIGR) MRI, we can objectively identify the necrotic cavitation and surrounding pericavitational regions within the lesion (Krishnamurthy et al., 2021). The pericavitational regions are defined as “damaged tissue within the lesion surrounding the core cavitation that may still contain living cell bodies” and have been observed in animal and *in vitro* models (Clarkson et al., 2010; Anderson et al., 2014; Burda and Sofroniew, 2014; Adams and Gallo, 2018; Joy and Carmichael, 2021). The pericavitational regions can be engaged using task fMRI, are functionally connected at rest to the remaining brain network and demonstrate evidence of residual blood flow to the lesion (Krishnamurthy et al., 2021). One downside to using only structural imaging to assess brain-behavior relationships is that language is processed in a network of brain regions that must work in concert, and language deficits can arise from disconnected or disrupted regions far away from the lesion. These disconnected regions can be identified by their changes in physiology (Metter et al., 1989). Therefore, to advance the field of stroke and aphasia rehabilitation, it is imperative to expand the current imaging models to not only relate anatomy to behavior but integrate both anatomy and physiology into behavior. To further understand the tissue health of chronic stroke lesions, we expand upon our previous findings and improve upon the detection of cerebral blood flow in chronic stroke lesions in PWA.

Cerebral blood flow (CBF) can be quantified non-invasively using a neuroimaging technology called pseudo-Continuous Arterial Spin Labeling (pCASL) which is a sub-class of arterial spin labeling (ASL) MRI methods available. Generally, the ASL experiment collects one set of images that contain signals from both tissue and blood compartments (the “control” image) and one set of images that contain signals from tissue and magnetically labeled blood to reduce the contribution of the blood signal (the “label” image). The difference between control and label images removes the tissue signal to reveal an image of pure blood signal (the “perfusion” image). To convert the perfusion image to physiological units (the “CBF” image), the perfusion image is further divided by a separately acquired proton density (M0) calibration image and modeled with sequence-specific factors such as post-labeling delay and physiological factors such as arterial blood T1. The consensus paper provides a comprehensive description of the technology (Alsop et al., 2015). Often these computations are achieved in native space–or the space in which the image was acquired–but most comparative studies require all participant CBF images to be normalized to template space. One current issue in the ASL MRI field is the standardization of processing steps to obtain absolute CBF maps (Pinto et al., 2020). For example, it is unclear whether some post-processing steps such as spatial smoothing should be applied in native or template space, if at all. Spatial smoothing is a process of spatially weighting the signal intensity from neighboring voxels to decrease the impact of instrumentation noise. There is a clear need for consistent post-processing options with a complete description of all post-processing steps, including spatial smoothing, to achieve absolute CBF quantification (Pinto et al., 2020). Steps such as spatial smoothing have an even greater impact when quantifying CBF in chronic stroke lesions due to the rapid transitions in microstructural damage (Burda and Sofroniew, 2014) and the impact on brain metabolism and corresponding CBF.

The objective of this project is to advance the ability of ASL MRI to reliably detect the CBF within the lesion and facilitate characterizing how lesion CBF relates to clinical factors relevant to aphasia. To accomplish this objective, we will optimize at what stage spatial smoothing should be applied in the ASL analysis pipeline. We hypothesize that the blood flow in chronic stroke lesions will reduce with increasing tissue damage. We also hypothesize that both stroke- and demographic-related factors will relate to lesion CBF. Finally, in an exploratory analysis, we will generate brain-behavior relationships between CBF and verbal learning or visuospatial learning and compare them to structural lesion-based brain-behavior maps.

## Materials and methods

### General procedures

Imaging and behavioral data from 14 English-speaking PWA (Table 1; age range 24–81 years old) who were >6 months post-left-hemisphere ischemic stroke (range of time since stroke 9–121 months) were analyzed for this study. Participants with a history of mental health disorders or other neurological disorders were excluded. Participants with hemorrhagic strokes were excluded due to the limitations of quantifying tissue damage in the presence of hemosiderin. Western Aphasia Battery Aphasia Quotient (WAB AQ) (range 27.4–80.6). The participants were asked to undergo an MRI session and a language assessment session which included the administration of the Western Aphasia Battery-Revised (WAB-R) (Kertesz, 2007), the Hopkins Verbal Learning Test-Revised (HVLT-R) (Benedict et al., 1998), and the Brief Visual Memory Test-Revised (BVMT-R) (Benedict et al., 1996). This study used raw scores from the HVLT-R and BVMT-R total recall, delayed recall, and recognition hits to compute brain-behavior relationships with CBF. This study was carried out in accordance with the recommendations of the joint review committee at Emory University and Atlanta Veterans Affairs Medical Center. All participants gave written informed consent in accordance with the Declaration of Helsinki.

**TABLE 1:** Participant demographic, aphasia, and lesion characteristics.

Sub	Age at scan	Sex	Months since stroke	WAB-AQ	Aphasia type	Lesion volume (mL)	Cavitation volume (mL)	% Cavitation
S01	51	M	24	59	Anomic	97,078	35,999	37.1
S02	59	M	38	65.9	Anomic	84,630	20,796	24.6
S03	50	M	85	75.3	Anomic	133,145	52,620	39.5
S04	71	F	24	67.1	Transcortical Motor	126,144	13,215	10.5
S05	61	M	121	74.5	Anomic	137,945	53,874	39.1
S06	24	F	23	55.3	Broca’s	133,655	38,924	29.1
S07	35	M	9	27.4	Broca’s	126,164	6,537	5.2
S08	47	M	44	75.6	Anomic	105,421	42,877	40.7
S09	43	F	49	80.6	Anomic	64,703	21,073	32.6
S10	81	F	60	74.1	Conduction	114,099	31,687	27.8
S11	73	M	55	79.6	Anomic	124,145	64,475	51.9
S12	45	M	14	78.6	Anomic	148,123	14,366	9.7
S13	50	M	70	59.4	Conduction	95,516	28,115	29.4
S14	60	M	9	52.4	Wernicke’s	132,319	44,429	33.6

### MRI acquisition

MRI scans were acquired on a 3T Siemens Prisma (Erlangen, Germany) using the body coil for radio frequency (RF) transmission and a 32-channel phased-array head coil for RF receiving. Two types of anatomical MRI scans were acquired on each subject: 1) a T1-weighted high-resolution anatomical image (T1-MPRAGE, TR = 2,530 ms, TE = 2.96 ms, TI = 1,100 ms, FA = 7°, isotropic resolution = 1 × 1 × 1 mm (Crosson et al., 2017), acquisition bandwidth = 130 Hz), and 2) a T2-weighted high-resolution anatomical image (T2-SPACE, TR = 3200 ms, TE = 285 ms, FA = 120°, isotropic resolution = 1 × 1 × 1 mm (Crosson et al., 2017), acquisition bandwidth = 700 Hz).

Pseudo–Continuous Arterial Spin Labeling (pCASL) MRI was collected to measure regional whole-brain cerebral blood flow (CBF) maps (2D ascending gradient echo EPI acquisition, 35 slices, slice thickness = 4 mm, 10% gap, matrix = 74 × 74, FoV = 220 × 220 mm^2^, in-plane resolution = 3 × 3 mm^2^, GRAPPA = 2, no Partial Fourier, acquisition bandwidth = 2,505 Hz, TR = 5,060 ms, TE = 13 ms, slice acquisition time = 37.5 ms, PLD = 2,200 ms, labeling duration = 1,500 ms, label offset = 90 mm). The pCASL method acquires interleaved control and label images, the subtraction of which yields a pure-blood signal that is directly proportional to CBF and can be mapped on a voxel-wise basis to obtain whole-brain regional blood flow information. An additional M0 scan was acquired with the same brain coverage as the pCASL scan, except for a longer repetition time (TR = 10 s) to allow for fully relaxed magnetization to remove proton density effects during CBF quantification.

### Estimation of tissue damage using TIGR maps

The workflow of calculating the Tissue Integrity Gradation via T2-weighted T1-weighted Ratio (TIGR) maps has been described previously (Krishnamurthy et al., 2021). Briefly, the user input includes a T1w and T2w image, as well as a binary lesion mask in native space. The T1w and T2w images are denoised (Coupe et al., 2008; Wiest-Daessle et al., 2008) and coregistered together via FreeSurfer’s boundary-based registration (Greve and Fischl, 2009). The values in each voxel of the T2w images are divided by the value of the corresponding voxel in the aligned T1w image (T2w/T1w). Each type of image (T1w and T2w) encodes unique signal information of the underlying tissue morphology. Taking the T2w/T1w ratio combines both types of information into one image to highlight the gradient of tissue damage within the lesion. To scale the T2w/T1w signals to a subject-specific value that can be compared across the entire cohort, the signal intensity is bounded by GM (lower bound:0.1; from the contra-lesional anterior grey matter ribbon eroded by one voxel) and CSF (upper bound:1.0; from the contra-lesional anterior lateral ventricle eroded by one voxel) and classified into nine “bins” between 0.1 (“least damaged”) and 1.0 (“most damaged”) to maintain comparability with binary lesion maps characterized by 0’s and 1’s that are often used in the field. Only the voxels within the user-defined lesion mask are classified into the tissue gradient “bins,” creating the final TIGR map used in group analysis. We further define all lesioned areas with a TIGR score of 1.0 as necrotic cavitation and all other regions (0.1–0.9) as surrounding pericavitational regions. To compare TIGR maps and CBF maps across PWA and generate brain-behavior relationships, the T2w/T1w ratio maps are spatially normalized to MNI template space using a “chimera” spatial normalization [described in the Supplementary Section of Krishnamurthy et al. (2021)]. The overlap of all participant’s lesions in the MNI template space is accomplished using afni’s 3dOverlap. The average of all participant’s TIGR scores in the MNI template space is accomplished using afni’s 3dmerge.

### Three analysis pipelines to compute CBF

The analysis of pCASL data is accomplished with in-house scripts using a combination of afni (version 22.2.10) and FSL (version 6.0.1) commands. The “no blur” pipeline uses the following analysis steps: 1) bulk-head motion correction is computed with afni’s 3dAllineate using 6 degrees of freedom. 2) The motion parameters are used to censor pairs of label and control images that contain motion of >0.7 mm and >5 degrees of rotation. A minimum of 32 pairs (out of a maximum of 40 pairs) were used for every participant’s dataset. 3) The label and control images were subtracted in native space to obtain the difference signal (=control-label) using afni’s 3dcalc and then averaged before conversion to physiological units. 4) To obtain CBF in physiological units, the difference signal (=control-label) and M0 image are combined with a single-compartment model to obtain units of mL/100 g/min (Buxton et al., 1998; Alsop et al., 2015). 5) The CBF map was then transformed into MNI space by registering to T1w space using FreeSurfer’s bbregister and subsequently applying the chimera warp into MNI space (FSL’s applywarp). All CBF results reported here are in 1 × 1 × 1 mm^3^ MNI space, which also conforms to the voxel size in the TIGR MRI map of tissue damage.

**FIGURE 1:**
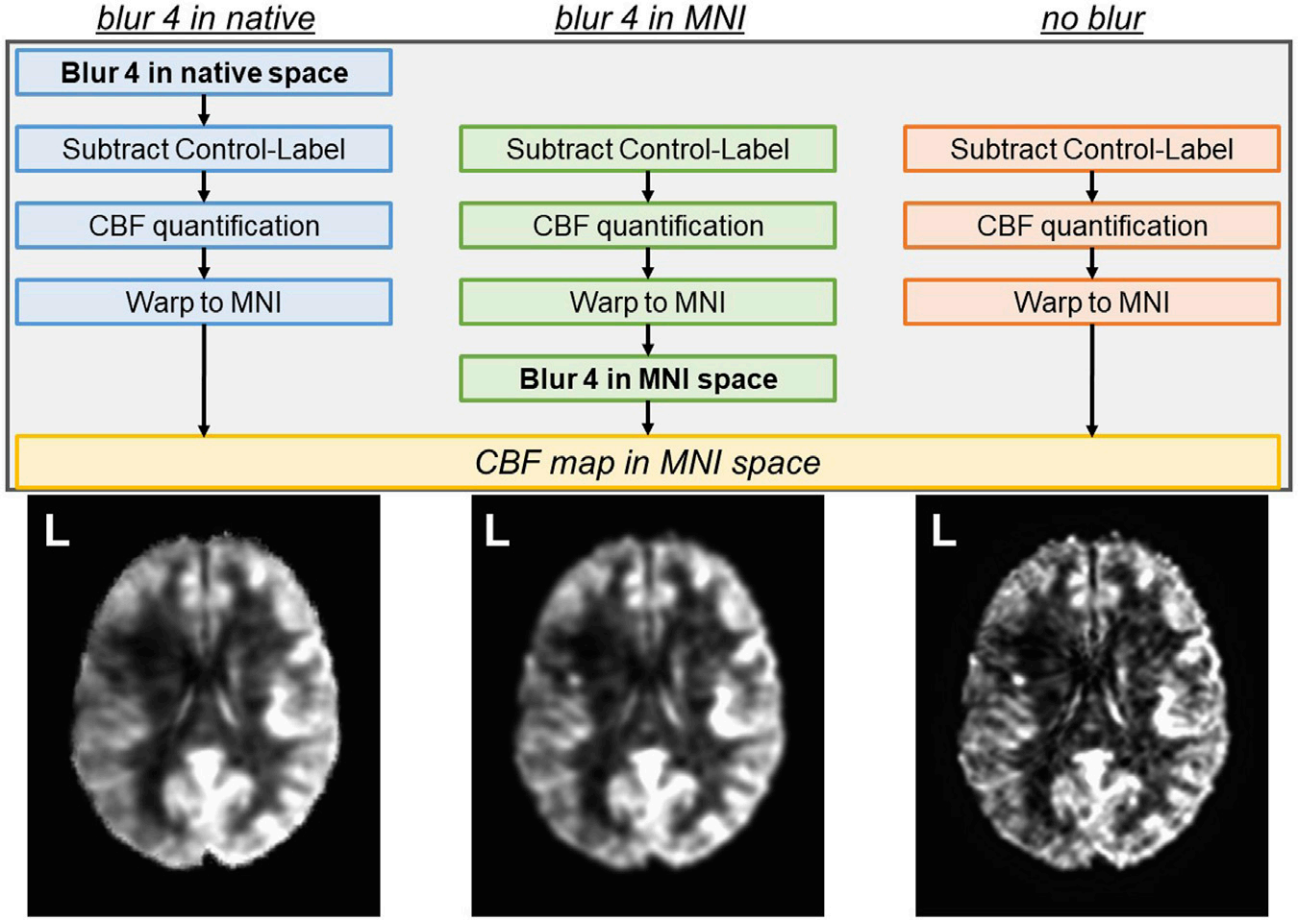
Three analysis pipelines to compute CBF were tested. The spatial smoothing in the “blur 4 in native” pipeline occurs in the native space, prior to the subtraction of control and label images. The spatial smoothing in the “blur 4 in MNI” pipeline occurs in the MNI space, and the “no blur” pipeline does not have spatial smoothing applied to the images. The resulting CBF map is always in MNI space. Average CBF maps from 14 participants (all left hemisphere lesions) for each analysis pipeline are shown as a reference.

The “blur 4 in MNI” analysis pipeline uses the output of the “no blur” pipeline and applies spatial smoothing (or blurring) within the brain using a 4 mm full-width-half-maximum (FWHM) Gaussian kernel (afni’s 3dmerge) to increase the signal-to-noise ratio (SNR). The “blur 4 in native” analysis pipeline introduces a 4 mm FWHM Gaussian kernel smoothing before the subtraction of control and label images in native space to increase the SNR before subtraction. The same transformations into T1w and MNI space computed for the “no blur” images were then applied to the “blur 4 in native” images to maintain comparability across all datasets. The general difference between the three analysis pipelines is depicted in Figure 1 with group average output images.

### Comparison between ASL analysis pipelines

To assess the impact of each of the ASL analysis pipelines on the non-lesioned CBF values in MNI space, we extracted a 15 mm radius sphere in the anterior cingulate cortex [ACC, MNI coordinates x = 0, y = 44 (anterior), z = 18 (superior)]. We ensured that the ACC 15 mm region of interest (ROI) did not overlap with any individual participant’s lesion. We then applied the grey matter (GM) and white matter (WM) segmentations computed on each participant’s T1w image using FSL’s fast algorithm (Zhang et al., 2001) to extract ACC GM and WM CBF values. The average CBF under each participant’s individual GM and WM CBF maps were extracted with afni’s 3dmaskave. A separate 15 mm spherical ROI outside of the brain and head was used to extract the standard deviation of the noise in the air. The following metrics were computed to assess the quality of the quantified CBF from each pipeline:

Signal-to-Noise Ratio (SNR)

$$SNR=\frac{\mu_{tissue}}{\sigma_{air}}$$ (1)

Coefficient of Variation (CoV)

$$CoV=\frac{\sigma_{tissue}}{\mu_{tissue}}$$ (2)

Grey matter to White matter Contrast-to-Noise Ratio (CNR)

$$CNR=\frac{\mu_{GM}-\mu_{WM}}{\sigma_{air}}$$ (3)

Where σ represents the standard deviation of the CBF within the ROI and μ represents the average CBF within the ROI.

### Relationship between TIGR-quantified tissue damage and CBF

We previously related perilesional regions (TIGR score = 0 within 10 mm of the lesion), low damage lesion regions (TIGR score 0.1–0.3), medium damage lesion regions (TIGR score 0.4–0.7), and high damage lesion regions (TIGR score 0.8–1.0) to decrease in cerebral blood flow in six participants (Krishnamurthy et al., 2021). We now expand upon this finding by leveraging the interpolated 1 × 1 × 1 mm^3^ CBF image to directly relate with the 1 × 1 × 1 mm^3^ TIGR map in 14 participants, allowing us to use the computed TIGR score rather than averaging over a range of TIGR scores. An ROI for each TIGR score was generated (0.1, 0.2, 0.3, … , 1.0) which is unique to each subject based on their lesion location, TIGR map profile, and ROI size. For each participant, an average CBF value was computed for each ROI.

To assess the relationship between CBF and tissue damage within the lesion, a linear regression was performed in JMP Pro16 (Cary, NC) for each participant. The fit of the linear model is reported with *R* ^2^ and F-statistic, with the corresponding *p*-value. To account for multiple tests being performed, significance was assessed using the Bonferroni corrected *p*-value of 0.01 divided by *N* = 14 participants.

Further, given that both demographic and stroke-specific factors may govern the amount of blood flow to the lesion, we completed an ANOVA in JMP Pro16 to test if factors (tissue damage) along with (time since stroke), (sex), (age), and cross terms (tissue damage*time since stroke), (tissue damage*sex), (tissue damage*age), (time since stroke*sex), (time since stroke*age), and (sex*age) explain CBF within the lesion. The results of the model are reported with F-statistic and subsequent t-tests are performed to determine which terms had a significant effect. We also tested a separate model that included WAB-AQ, as the aphasia quotient can be used in the clinical setting.

### Multivariate brain-behavior relationships

We recently established that whole-brain task-fMRI data can be used to quantify brain-behavior maps in PWA (Song et al., 2023). Based on these promising results, we built upon this multi-variate framework by using whole-brain CBF maps. We assessed the feasibility of quantifying brain-behavior relationships between whole-brain CBF maps and either HVLT-R or BVMT-R behavioral measures using LESYMAP’s sparse canonical correlation analysis (sccan) (Pustina et al., 2018) thresholded at *p* < 0.05. Because LESYMAP’s sccan models all voxels simultaneously, no multiple comparison corrections are necessary. To determine if whole-brain CBF maps can capture brain-behavior results above and beyond structural imaging modalities, we show results for TIGR maps and CBF maps, all in MNI template space to facilitate group statistics and comparison across methodologies. To account for the effects of lesion on the CBF values, we covaried two lesion-derived metrics from each voxel only if the relationship was significant: 1) lesion volume and 2) cavitation volume. There were no other covariates such as age entered into the modeling of the brain-behavior relationship. Due to the whole-brain nature of CBF data, a whole-brain mask was generated by overlapping binarized anatomical brain masks from each participant (afni 3dOverlap) and thresholded at a minimum of *N* = 10 participants represented in each voxel (afni 3dcalc). The k-fold cross-validation for sccan was set to 7 and the output results were clustered at 1,000 contiguous voxels.

## Results

### General results

**FIGURE 2:**
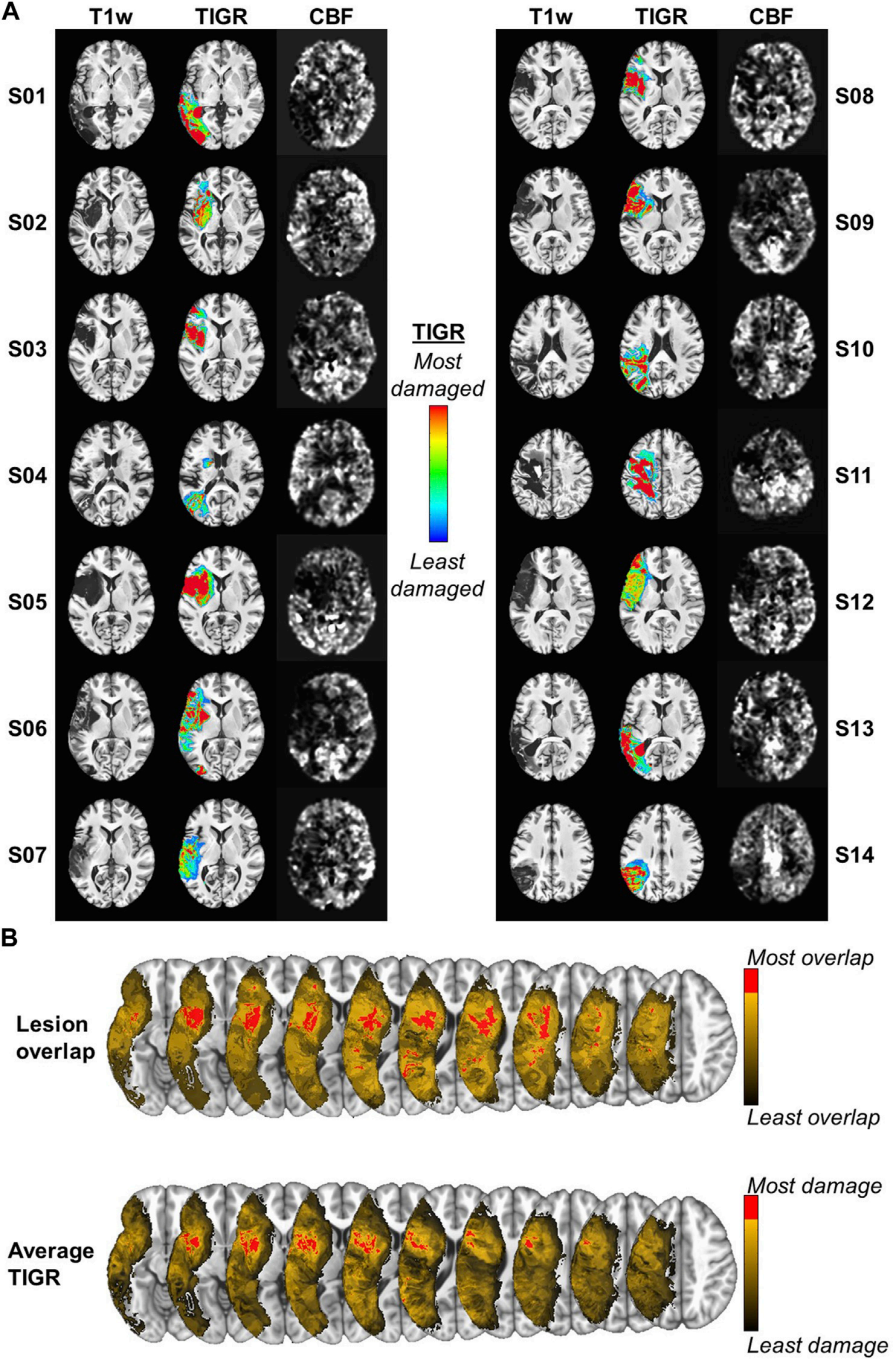
**(A)** The T1w, TIGR MRI, and CBF map from each individual participant, showing the heterogeneity of lesion location, tissue damage, and CBF maps. **(B)** The maximum lesion overlap and the maximum average TIGR scores are in adjacent, but only partially overlapping, areas. Note: Left is left in the images.

PWA characteristics are shown in Table 1. All 14 participants were ensured to have good-quality T1w, T2w, and pCASL images via careful image pre-processing and quality control. The lesion location was heterogeneous across the MCA territory and the CBF visually decreased in their respective lesioned areas (Figure 2A). The false-color TIGR map ranges from blue (0.1, least damaged) to red (1.0, most damaged) and estimates the degree of tissue damage in the lesioned area (Figure 2A). Of the 14 participants, the maximum number of lesions that overlapped in any given region was 9 but did not overlap with the maximally damaged regions as quantified by TIGR (Figure 2B). The red portion of the colorbar in Figure 2B represents the highest 80% of the maximum overlap or average tissue damage. The lesion overlap is greater in white matter regions, whereas the greatest average tissue damage is in gray matter regions (Figure 2B).

### Comparison of ASL analysis pipelines in intact brain regions and the whole lesion

**FIGURE 3:**
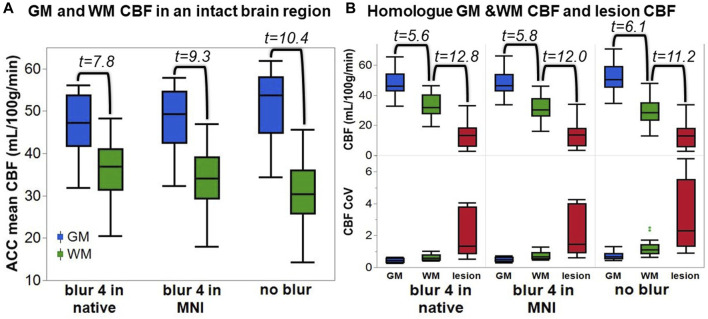
The quantified CBF from each ASL analysis pipeline. **(A)** The anterior cingulate cortex (ACC) GM and WM CBF. The number represents the t-statistic of a paired t-test between tissue types. **(B)** Top row: The CBF of the lesion and its homologue GM and WM. The number represents the t-statistic of a paired t-test between regions. Bottom row: The CoV of the CBF across the same tissue regions for each analysis pipeline.

The average ACC GM CBF was 46.7 ± 7.7 mL/100 g/min for blur-4-in-native, 47.7 ± 8.0 mL/100/min for blur-4-in-MNI, and 51.2 ± 8.5 mL/100 g/min for no-blur ASL analysis pipelines. The average ACC WM CBF was 36.2 ± 7.4 mL/100 g/min for blur-4-in-native, 33.9 ± 7.7 mL/100 g/min for blur-4-in-MNI, and 30.5 ± 8.5 mL/100 g/min for no-blur ASL analysis pipelines. An ANOVA testing the effect of ASL analysis pipeline and tissue type on average CBF values was significant [F (3,80) = 23.66, *p* < 0.0001], but indicated that only the tissue type had a significant effect on average CBF values (F = 70.87, *p* < 0.0001), while the ASL analysis pipeline did not have a significant effect on average CBF values (F = 0.06, *p* = 0.95). There is, however, a small effect of the ASL analysis pipeline on GM-WM CNR [F (2,39) = 4.64, *p* = 0.02], indicating that the no-blur ASL analysis pipeline provided a greater CNR (2.8 ± 1.3) than the blur-4-in-MNI (CNR = 2.1 ± 1.2) and the blur-4-in-native (CNR = 1.5 ± 0.9). This translates to the increased pairwise t-statistic in GM and WM (Figure 3A), where blur-4-in-native has pairwise GM-WM difference with t (13) = 7.8 (*p* < 0.0001), blur-4—in-MNI has a pairwise GM-WM difference with t (13) = 9.3 (*p* < 0.0001), and no-blur has the greatest pairwise GM-WM difference with t (13) = 10.4 (*p* < 0.0001). A significant decline with age in ACC GM CBF was detected (t = −2.2, *p* = 0.04), but not in ACC WM CBF (t = 0.5, *p* = 0.60).

The lesion CBF is significantly lower than the homologue WM CBF (t = 11.2–12.8, *p* < 0.0001), which is remarkably lower than the homologue GM CBF (t = 5.6–6.1, *p* < 0.0001), regardless of ASL analysis pipeline (Figure 3B). The CoV of the CBF in the lesion area is greater than in GM or WM, suggesting that more transitions in CBF are captured within the entire lesion ROI compared to other tissue types. Because not all lesioned tissue is equal, and some regions may have more or less blood flow due to the degree of tissue damage, it supports our expectation that a relationship between tissue damage and CBF may be detectable.

### Assessing the relationship between lesion CBF and tissue damage

**FIGURE 4:**
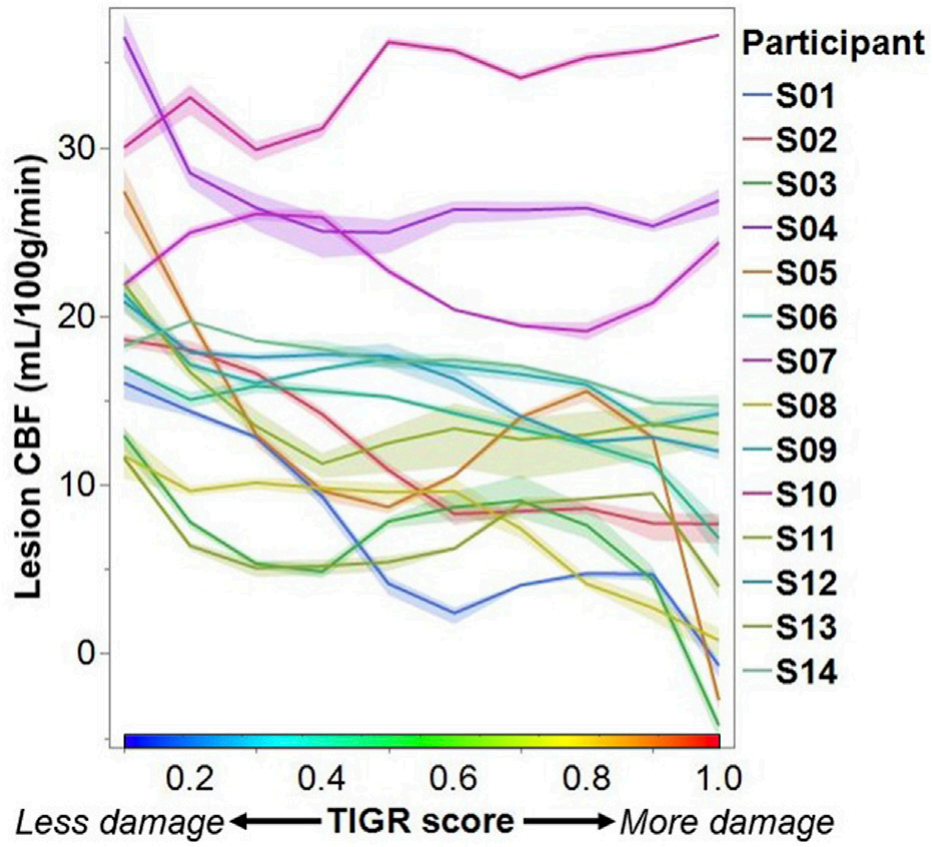
Each participant’s lesion CBF at each TIGR score. Each line represents the average CBF computed from all three ASL analysis pipelines. The error envelope is constructed using the maximum and minimum CBF computed from all three pipelines.

To assess the impact of tissue damage on blood flow, we plotted the average lesion CBF against the TIGR score for each participant. Most participants tend to have a higher CBF in less damaged tissue and a lower CBF in more damaged tissue (Figure 4). To assess if the relationship between CBF and TIGR is significant, linear regression was applied to each participant’s dataset for each analysis pipeline. As seen in Table 2, 6 out of 14 participants had a Bonferroni-corrected significant relationship between CBF and TIGR. Further, an additional 5 participants had non-Bonferroni corrected significant relationships between CBF and TIGR. Only one participant (S13) did not indicate any relationship between CBF and TIGR score. Further, 12 out of 14 participants showed the expected negative relationship between CBF and TIGR as indicated by the column “sign of the slope” in Table 2. One participant showed a significant positive relationship between CBF and TIGR. It is also evident in Figure 4 that there is a lot of variability in the lesion CBF that is not explained by tissue damage. For example, there is variability in the CBF of the least damaged regions (TIGR = 0.1), which ranges from 11–36 mL/100 g/min. Therefore, in the next section, we introduce a model with more explanatory factors to further describe the CBF within the lesion.

**TABLE 2:** The statistics describing the linear relationship between CBF and TIGR for each subject (Sub) and each analysis method: blur-4-in-native, blur-4-in-MNI, and no-blur. The * in the *p*-value column indicates significance at a Bonferroni corrected *p* = 0.01. The sign of the slope column indicates if the relationship is negative (−) or positive (+). The bolded numbers correspond to the values with a * (significance at a Bonferroni corrected *p* = 0.01).

Sub	Blur 4 in native			Blur 4 in MNI			No blur			Sign of the slope
	*R* ^2^	F (1,8)	*p*-value	*R* ^2^	F (1,8)	*p*-value	*R* ^2^	F (1,8)	*p*-value	
S01	0.84	43.34	0.0002*	0.82	37.70	0.0003*	0.78	28.67	0.0007*	-
S02	0.86	48.30	0.0001*	0.88	56.39	<0.0001*	0.90	73.98	<0.0001*	-
S03	0.45	6.52	0.03	0.46	8.82	0.03	0.29	3.34	0.10	-
S04	0.35	4.27	0.07	0.36	4.57	0.07	0.25	2.65	0.14	-
S05	0.50	7.99	0.02	0.48	7.27	0.03	0.46	6.73	0.03	-
S06	0.82	36.22	0.0003*	0.81	33.69	0.0004*	0.75	24.05	0.001	-
S07	0.23	2.40	0.16	0.18	1.75	0.22	0.17	1.67	0.23	-
S08	0.85	45.44	0.0001*	0.81	34.52	0.0004*	0.82	37.45	0.0003*	-
S09	0.62	13.29	0.007	0.64	14.36	0.005	0.63	13.56	0.006	-
S10	0.71	19.63	0.002	0.66	15.70	0.004	0.57	10.44	0.01	+
S11	0.44	6.27	0.04	0.34	4.05	0.08	0.24	2.59	0.15	-
S12	0.94	127.32	<0.0001*	0.92	90.70	<0.0001*	0.86	50.82	<0.0001*	-
S13	0.02	0.16	0.70	0.01	0.07	0.80	0.01	0.07	0.80	
S14	0.87	55.28	<0.0001*	0.90	75.63	<0.0001*	0.77	26.65	0.0009	-

### Modeling the effect of tissue damage, time since stroke, age, and sex on blood flow to the lesion

The CBF within the lesion was modeled with a group-level ANOVA to further account for the variability by introducing stroke-related factors tissue damage (from TIGR) and time since stroke, and demographic-related factors age and sex, as well as their cross terms. The CBF output from each of the three ASL-analysis pipelines was modeled in separate ANOVAs and the factor outputs are summarized in Table 3. The significant factors include tissue damage, time since stroke, sex, age, and cross-terms age-by-sex and time since stroke-by-age. These factors were significant, regardless of the analysis pipeline, but the best fit of the lesion CBF was provided by the blur-4-in-MNI ASL-analysis pipeline (*F* = 55.62, *p* < 0.0001). Although the no-blur pipeline provided the greatest GM-WM contrast in intact brain regions (Figure 3), the resulting ANOVA for the no-blur CBF provided the lowest fit as indicated by the F-statistic (Table 3). Because WAB-AQ is also a clinical (stroke-related) factor, we tested the addition of WAB-AQ to the model but found that no terms with WAB-AQ remained significantly related to lesion CBF. Therefore, we only consider the model without WAB-AQ factors.

**TABLE 3:** The ANOVA model output for each ASL analysis pipeline. The numbers indicate a t-statistic, except the bottom row, which is an F-statistic. The red box indicates that blur-4-in-MNI produces the CBF values that are best described by the model. Note: For each model factor, **** indicates *p* < 0.0001, *** indicates *p* < 0.001, ** indicates *p* < 0.01, * indicates *p* < 0.05, and N/S is not significant. The bolded numbers indicate the greatest t or F statistic in that row (if significant).

Model factor	Blur 4 in native	Blur 4 in MNI	No blur
sex	11.83****	11.19****	9.96****
age at scan	9.40****	10.47****	9.25****
age at scan × time since stroke	8.37****	8.91****	8.12****
age at scan × sex	6.80****	6.34****	5.74****
tissue damage (TIGR)	−4.72****	−5.16****	−5.04****
time since stroke	−4.11****	−3.95***	−3.25**
tissue damage × sex	2.32*	2.03*	1.93^N/S^
tissue damage × age at scan	1.55^N/S^	1.59^N/S^	1.44^N/S^
tissue damage × time since stroke	−0.96^N/S^	−0.96^N/S^	−0.84^N/S^
time since stroke × sex	−0.34^N/S^	−0.07^N/S^	0.25^N/S^
*F(10,129) =*	54.90****	55.62****	45.36****

**FIGURE 5:**
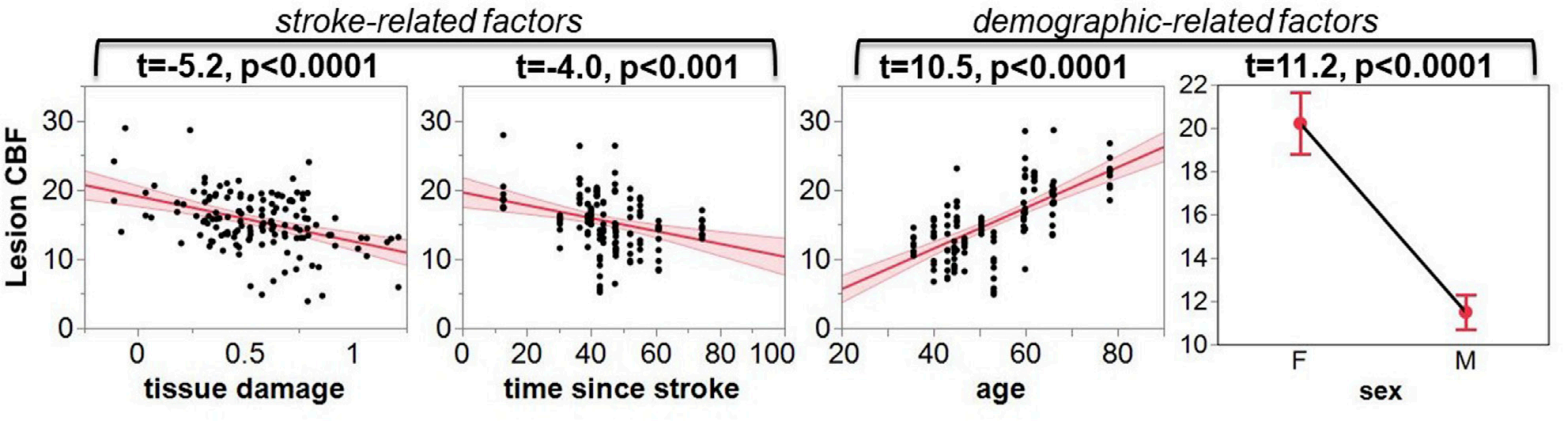
The leverage plots for the ANOVA model output. The lesion CBF is significantly associated with stroke-related factors of tissue damage and time since stroke, and demographic-related factors of age and sex.

As indicated by Figure 5, more regional tissue damage within the stroke lesion is related to a lower CBF within that region (defined by the TIGR maps). Further, if more time has elapsed since the stroke, the lesion CBF is also lower. The model also suggests that the lesion CBF is higher in older participants compared to younger, which is opposite to the intact ACC GM CBF. The model also identified that Females tend to have a greater lesion CBF compared to Males. Finally, there was an interaction between the two demographic factors age-by-sex, but more interestingly, the model also indicated a significant interaction between stroke and demographic-related factors age-by-time since stroke. Because the blur-4-in-MNI CBF output resulted in the best fit of stroke and demographic-related factors, this CBF value is further graduated to determine if brain-behavior relationships could be identified with CBF maps.

### Multivariate brain-behavior relationships

If a voxel’s CBF value across the group was significantly related to either lesion or cavitation volume, the relationship was covaried out to reduce any confounds in the identified brain-behavior relationships. No significant relationships between lesion volume or cavitation volume were identified with TIGR maps and therefore not regressed out.

**FIGURE 6:**
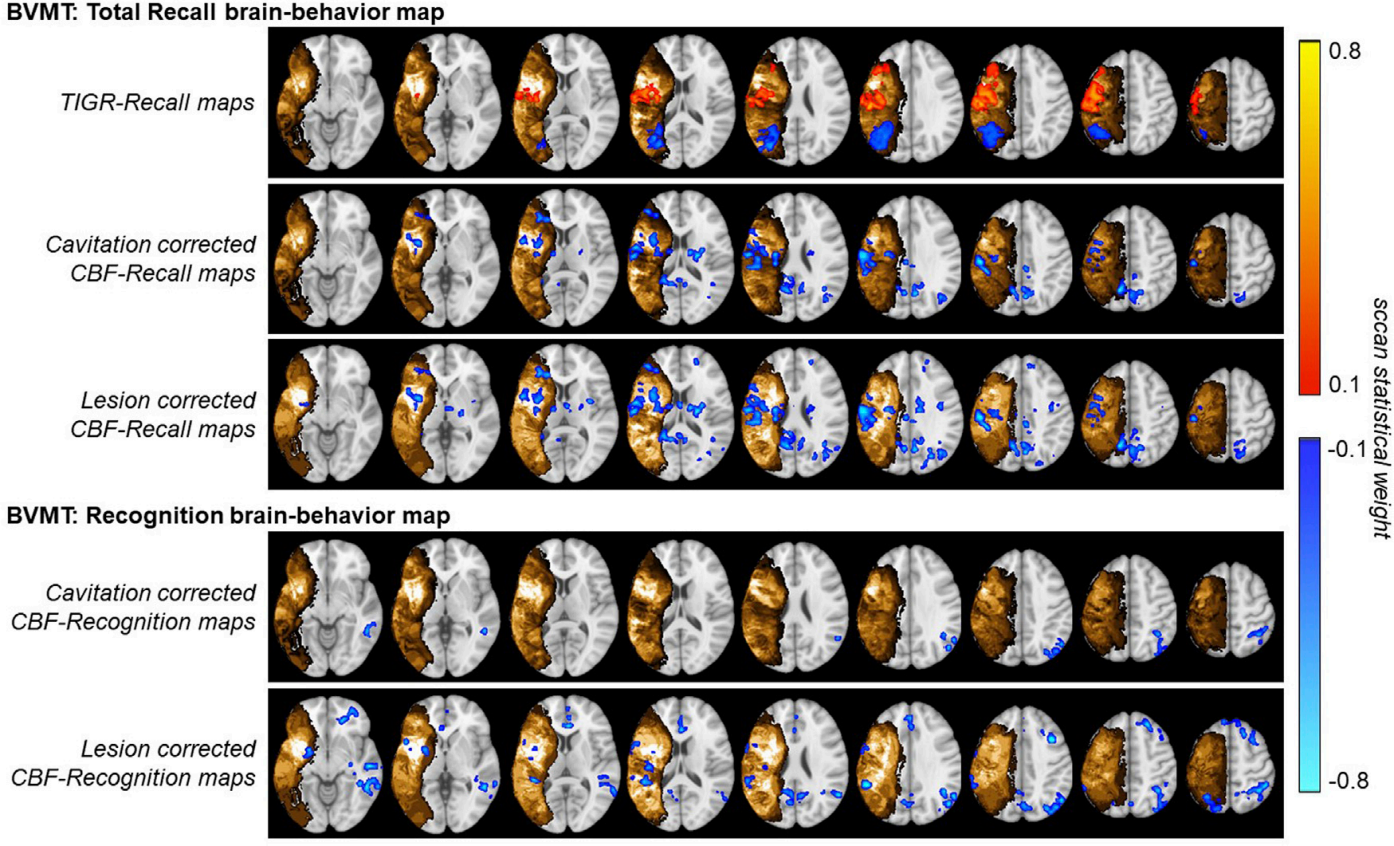
Multivariate brain-behavior maps using TIGR MRI and either cavitation-corrected or lesion-corrected cerebral blood flow (CBF) for BVMT total recall and BVMT recognition. The gold indicates either mean TIGR (cavitation corrected) or lesion overlap (lesion corrected). The red and blue indicate regions of significant multivariate brain-behavior relationships.

Significant multivariate brain-behavior relationships (thresholded at *p* < 0.05) were found for BVMT total recall and BVMT recognition for both cavitation and lesion-corrected CBF data (Figure 6). TIGR maps resulted in significant brain-behavior relationships with BVMT total recall. The cavitation-corrected CBF maps show fewer brain-behavior areas than the lesion-corrected CBF brain-behavior maps, although the two input data tend to agree on the areas where both are significant. The results in Figure 6 show that CBF-derived brain-behavior maps for BVMT–total recall are distinct from the brain-behavior maps for BVMT–recognition, suggesting that whole-brain CBF maps can generate behavior-specific information. Further, the TIGR relationship with BVMT total recall shows one overlapping and one unique area compared to the CBF relationships with BVMT total recall, suggesting the structural and functional modalities are complementary. The BVMT total recall maps include the anterior thalamus and retrosplenial cortex, both of which are associated with spatial-memory-related behavior (Vann et al., 2009). Conversely, the BVMT–recognition shows areas of the default mode network and the right executive function network, which are involved in decision-making (Sridharan et al., 2008). Finally, the HVLT behavior did not result in significant brain-behavior maps using LESYMAP’s sccan multivariate analysis for either TIGR or CBF imaging inputs.

## Discussion

The results of this study demonstrate that CBF is detectable in chronic stroke lesions, suggesting that the pericavitational regions may be viable and can be engaged and targeted during aphasia and stroke rehabilitation. We recommend that at least in chronic stroke datasets, spatial smoothing should be applied in the MNI template space during the CBF analysis to increase the grey matter and white matter contrast while maintaining sensitivity to changes in tissue damage. We showed for the first time that the CBF in chronic stroke lesions decreases with increasing tissue damage as quantified by TIGR MRI. Further, we showed that demographic and stroke-related factors also influence the lesion blood flow, suggesting that individualization of stroke intervention strategies is a priority to achieve optimal treatment outcomes. Finally, using an advanced multivariate approach, for the first time, we demonstrated that whole brain CBF in PWA is related to visual-spatial learning and memory and can serve as complementary information to lesion-based brain-behavior maps.

### Comparison of ASL analysis pipelines

Measuring cerebral blood flow within chronic stroke lesions is challenging because of the small ASL signal and additional reductions caused by tissue damage. However, we have shown that standard pCASL MRI can effectively measure CBF within these lesions. We improved the detection sensitivity of changes in CBF in rapidly transitioning tissue damage by optimizing the step in which spatial smoothing is applied within the ASL analysis pipeline. Our approach increases the sensitivity to differences in tissue types, including grey matter and white matter, as well as quantifying tissue damage using the TIGR score. It is well accepted that GM has a higher CBF than WM and previous studies have determined that pCASL MRI can reliably detect WM perfusion (van Osch et al., 2009). Therefore, we first assessed the effects of spatial smoothing on the non-lesioned ACC and determined that the GM-WM differences are detectable regardless if smoothing is applied in native space, MNI template space, or not at all. However, there was an increase in tissue contrast, when smoothing was applied in MNI template space compared to native space, but the highest GM-WM CNR was found if no smoothing was applied. Given these results, we thought that no smoothing would provide the best snapshot of lesion CBF, however, when modeling the CBF with demographic and stroke-related factors (Table 3), it became apparent that spatial smoothing in MNI template space was the most appropriate.

As is well known from the functional and physiological MRI literature, spatial smoothing provides stability to the regional MR signal, generally benefitting the ASL perfusion signal (Wang et al., 2005; Fazlollahi et al., 2015) and minimizing the anatomic variability in group-level analyses (Scouten et al., 2006; Mikl et al., 2008). However, there are also some negative outcomes of spatial smoothing such as reductions in tissue CNR (Molloy et al., 2014), loss in precision of cluster extent (Hagler et al., 2006), and increased Type-1 errors in the estimation of group-level significant maps (Vul et al., 2009). While we observed the expected decrease in the tissue CNR, such a limitation should be evaluated within the context of study goals. For clinical neuroradiological evaluation of CBF maps at the individual level, perhaps tissue CNR becomes more important. Instead, for group-level investigational research studies, we show that the improved relationship between stroke and demographic-related factors outweighs the reduced CNR. Although the tissue damage transitions in the TIGR images can be as rapid as 1–2 mm, possibly limited by the resolution of the T1w and T2w images, blurring the CBF maps still improved the overall relationship with tissue damage and other factors. It will be of interest to repeat this study at the higher field strength of 7T, where submillimeter resolution ASL data can be collected (Zuo et al., 2013) and the SNR is 3-fold compared to 3T (Shao et al., 2021). Perhaps due to these inherent signal improvements, smaller transitions in the stroke lesion CBF signal can be detected at 7T. However, technical limitations for implementing ASL at 7T such as specific absorption rate (SAR) limits and the ability to achieve reasonably strong labeling at a reasonably large labeling-plane offset may preclude a widespread adaptation of TIGR and ASL data collection in stroke participants and will likely serve as a validation of the present findings. Instead, translating the current approach to wide-bore research and clinical systems with lower gradient amplitudes may help the translation of the approach into the clinic, particularly for post-stroke individualization of rehabilitation approaches. Finally, the impact of spatial smoothing and smoothing kernel size in MNI space and its effect on the extent of detected cluster size and Type-1 errors is important but was not the focus of the current study. Future work should consider advancing the stroke ASL analysis pipelines to investigate these questions using larger datasets.

Another challenge to CBF quantification is the estimation of tissue proton density (M0) to calibrate the perfusion signal. We used a separately acquired M0 image to calibrate the perfusion data on a voxel-by-voxel basis, rather than using the whole brain difference signal or intact region as the calibration reference signal, as has previously been used to reduce inter-subject variability (Aslan and Lu, 2010). We hypothesize that the M0 voxel-by-voxel normalization may be the most appropriate for stroke brains because a whole-brain average will be influenced by lesion size and location, possibly introducing more inter-subject variability, and the intact control region may be affected by changes in blood flow distal to the lesion (i.e., diaschisis), further introducing more inter-subject variability. Future work should compare and contrast the different CBF normalization techniques.

### The relationship between lesion CBF and tissue damage

We assessed the relationship between lesion CBF and tissue damage using linear regression. A significantly decreasing CBF with increasing tissue damage was detected in 6 out of 14 participants at a Bonferroni corrected *p* = 0.01 and in an additional five participants at a non-Bonferroni corrected value (Table 2) suggesting that this relationship will be detectable in most chronic ischemic stroke lesions. In one participant, who is characterized as a 50-year-old Male, 70 months post-stroke, with Conduction aphasia, no relationship between CBF and tissue damage was found; however, this was also the participant with the lowest CBF value at a TIGR score of 0.1 (least tissue damage). Therefore, the detectability of further tissue-damage-related CBF decreases may be minimal for this participant. Further, the CBF for this participant does decrease between TIGR scores 0.1 and 0.6, but then rises again to peak at TIGR score 0.9 and then falls to a minimum in the cavitation (TIGR score 1.0). Indeed, the rise of CBF at a mid-level TIGR score is observed in approximately half of the participants (Figure 4), indicating that there may be a tissue-type transition. We speculate that perhaps the lower TIGR scores of 0.1–0.5 (less damage) are mapped to white matter, while TIGR scores of 0.6–0.9 (more damage) are mapped to grey matter. This would explain the “jump” in CBF mid-way through the tissue damage scores because GM always has higher CBF than WM but is now confounded by the amount of damage impacting the tissue. This does not mean that GM is necessarily always more damaged than white matter, but may perhaps stem from the inherent differences in tissue properties (cell type and morphology, tissue T1 and T2, etc.) that are a latent confound to TIGR tissue damage classification. The cavitation (TIGR score 1.0) is mapped indiscriminately to either tissue type. It is worth noting that the ratio of T1w to T2w anatomical images is known, rightly or wrongly, in other contexts as the “myelin map” (Glasser and Van Essen, 2011; Boaventura et al., 2022) due to its strong association with myelin content in the non-lesioned brain. The ratio of T1 to T2 in myelin maps or T2 to T1 in TIGR maps is a representation of microstructure differences across brain regions–which, in a healthy brain can grossly be attributed to regional differences in myelination. In stroke lesions, or most neurological diseases, it is not so simple because the microstructural changes are so vast and varied that the change in the ratio cannot be attributed to a single cell type. Empirical data to further investigate these observations does not yet exist, and will likely rely on models to determine the previous individual sulcal and gyral patterns of the lesioned tissue. These models may also take into account atrophy and pressure-related changes in sulcal patterns. All other participants showed a significant reduction in CBF with increasing tissue damage, as hypothesized, except for one, which may be due to the pCASL MRI sequence, which is discussed later in the Limitations section.

### Lesion CBF relates to stroke and demographic factors

The model output showed that CBF within the lesion was significantly described by demographic factors “age” and “sex” and stroke-related factors “time since stroke” and “tissue damage.” CBF is a complex measure of vascular and metabolic supply to the tissue (Drake and Iadecola, 2007), and incorporating all of these factors in the model allows for an improved understanding of clinically-relevant factors that are important for diagnosis and treatment planning (Table 3; Figure 5).

It is well accepted that CBF reduces with age in healthy participants (Mokhber et al., 2021), which we also detected in the intact ACC GM ROI establishing the validity of our analysis pipeline. However, the model result showing that lesion CBF is more preserved in older individuals compared to younger individuals is somewhat counterintuitive (Figure 5). It may be that the global aging-related decline in brain connections results in a reduced impact on blood flow to the lesion, or may have an impact on post-stroke vascular dynamics. While it is encouraging that our results reflect these exciting findings, empirical evidence of such a relationship does not yet exist and more sophisticated multi-modal work is needed to characterize the lesion physiology and microstructure in humans.

The model also showed that Females have more preserved blood flow to the lesion than Males, but this finding conforms to the bulk of the literature on healthy aging, where cortical CBF in Females is greater than in Males, at least at an age less than 65 (Aanerud et al., 2017). The significant age-by-sex interaction term further indicates that the lesion CBF may be sensitive to Male-Female CBF differences that change with age. However, the small sample size of Females (*N* = 4, age 24–81) compared to Males (*N* = 10, age 35–73) strongly indicates that this finding needs to be replicated with a much larger cohort with a wide range in age and balanced representation of Males to Females.

The stroke-related factor “time-since-stroke” showed that lesion CBF declines as more time passes since the stroke. This may indicate that metabolic and vascular declines are perhaps still occurring in the chronic stages of stroke in the lesioned area, plausibly due to the loss of neural cells due to lack of blood supply. This is an interesting finding, as subcortical structures downstream from the lesion do not change their volume in the chronic stages (Krishnamurthy et al., 2020), indicating that these are subtle metabolic and vascular processes that may occur locally and closer to the lesion. Furthermore, the significant age-by-time since stroke interaction further indicates that the rate of decline in CBF after stroke may depend on the age at which the stroke occurred. This is one of the first pieces of evidence that aging-by-disease interactions can be quantified to help in individualized patient treatment planning.

The data indicated that lesion CBF decreases significantly with increasing tissue damage (Table 2; Figure 4). This result was reinforced in the model that accounted for both stroke and demographic-related factors (Table 3; Figure 5). Therefore, if TIGR MRI indicates that a region of the chronic stroke lesion is pericavitational, it is likely that the damaged-but-intact tissue is viable and can be reengaged with targeted rehabilitation.

### Brain-behavior relationships

When examining the relationship between the brain and behavior, researchers have relied on lesion information for over 200 years (Baldo et al., 2022). Although powerful and highly informative, this approach limits the search for relationships to only the areas where lesions occur. In PWA, that usually means only the left hemisphere middle cerebral artery (MCA) territory is inspected for brain-behavior relationships. Instead, using whole-brain cerebral blood flow maps with CBF that is detectable in the lesioned areas allows the entire brain now to become available when assessing functional and physiological brain-behavior relationships. First, we will examine the lesion-level brain-behavior relationships and then extend the results into the CBF-based brain-behavior relationships.

We previously showed that brain-behavior maps extracted with TIGR show similar brain-behavior maps compared to binary lesions (Krishnamurthy et al., 2021), but may have more statistical power due to the continuous nature of TIGR compared to the binary “all-or-none” nature of lesions maps. Therefore, given the small sample size of *N* = 14 participants, we chose to evaluate lesion-based brain-behavior maps using TIGR as the input. The BVMT total recall related significantly to two regions of the group-level TIGR maps: 1) a left frontal region encompassing primary sensory-motor, dorsal pre-motor, and dorsal lateral prefrontal cortices, as well as the posterior insula, and 2) a left parietal region encompassing supramarginal gyrus and angular gyrus. The frontal region is positively related to tissue damage, which indicates that damage to these regions predicts better visuospatial function. This result is counterintuitive because damage to any brain region will likely result in some level of worse behavioral outcome. However, it may be that damage to the frontal regions indicates a lower probability of damage to the parietal regions, which instead shows a negative relationship between tissue damage and BVMT total recall. In PWA, the left inferior parietal lobule (IPL) is often associated with language processes, but in the case of the visuospatial learning captured by the BVMT total recall, it may be that this finding suggests that visuospatial learning is processed in both the right and left parietal cortices because functions such as new learning are not clearly localized (Goodglass et al., 1979). The clues to this can be gathered from the CBF-derived brain-behavior maps.

The CBF-derived brain behavior maps show multiple distinct areas, both within the lesion and distal to the lesion. The CBF-derived brain-behavior region overlaps with the TIGR-based frontal region, while the TIGR-based parietal brain-behavior region does not overlap with the CBF-derived region. Therefore, the left parietal involvement identified with only the TIGR information may represent complementary information. It is not clear why the CBF-related regions do not overlap with all TIGR-related regions, especially since the TIGR ad CBF scores are linearly related. However, other regions outside of the lesion are highlighted when using CBF: specifically the right IPL. Therefore, the L-IPL from TIGR and R-IPL from CBF together point to both hemispheres’ involvement in BVMT total recall, showing that visuospatial learning is related to the baseline blood flow to the right parietal areas (as traditionally implicated in visuospatial tasks) as well as the left parietal areas. The BVMT recognition task, which recalls which shapes were encountered, has more right lateralization in the CBF brain-behavior maps. This interpretation would have been difficult when assessing lesion-derived brain-behavior maps in the absence of the CBF-derived maps because the additional network information is missing when confined to only the lesion overlap areas. We recommend that the brain-behavior mapping field go beyond lesion-based maps and start incorporating whole-brain functional and physiological maps as inputs. Both types of maps may agree on some regions but seem to also provide unique and complementary information that together helps improve our understanding of the brain and determine targets of intervention.

We did not identify significant brain-behavior relationships with verbal learning as measured with the HVLT-R, although the reasons for this are not clear as the behavior was well distributed between low and high values, and the lesion locations were also distributed across the MCA territory. Perhaps the CBF maps did not show a relationship to HVLT-R because verbal learning is more confined to the left hemisphere compared to visuospatial learning. However, TIGR MRI also did not relate to HVLT-R which was disappointing as verbal learning in PWA is a predictor of rehabilitation success (Dignam et al., 2017), and identifying the hubs of the brain network involved in verbal learning may have been informative for treatment planning.

In this report, we also assessed the effects of covarying the lesion volume versus the cavitation volume from the CBF maps before computing the brain-behavior maps. Both cavitation volume and lesion volume had areas of significant correlation with CBF, and both cavitation-corrected and lesion-corrected CBF resulted in significant brain-behavior maps with similar regions (Figure 6). Based on the number of significant regions captured by each modality, we recommend that the field continue to correct by lesion volume, not by cavitation volume. The cavitation-corrected CBF, at least at this small sample size, seems to lose some statistical sensitivity relating to behavior (both subcortical and frontal regions are missing when correcting by cavitation size). Although the cavitation represents the part of the lesion that is fully damaged, it may be that the distal blood flow is more impacted by the size of the lesion, regardless of how much potentially viable tissue is still present in the lesion.

Finally, all brain-behavior results using CBF show a negative relationship, indicating that greater blood flow to those brain regions predicts a worse behavioral outcome. Such a result, though counterintuitive, is not beyond the realm of possibility. A theoretical, yet empirically elusive, construct in the stroke literature is the concept of diaschisis (Carrera and Tononi, 2014)—defined as “neurophysiological changes that occur distant to a focal brain lesion.” It may be that loss of input and output to a region distal to the lesion may cause an increase in blood flow due to a reduction in inhibitory tone (Blicher et al., 2015), which together (i.e., GABA and CBF) influence cognitive decline (Krishnamurthy V. et al., 2022; Krishnamurthy L. C. et al., 2022). Therefore, an increased CBF distal to the lesion may indicate a failure of the network and lead to worse behavioral outcomes.

### Limitations

This study is the first of its kind to assess the CBF in chronic stroke lesions and relate to the underlying tissue damage of the lesion. While the study has many novel discoveries, there are a few limitations that also need to be highlighted. First, the study sample size of *N* = 14 chronic stroke participants is small and therefore should be viewed strictly as a proof-of-principle. Particularly the model output and the brain-behavior relationships require further testing with larger cohorts, as these statistical tests usually require dozens of participants to identify significant relationships (Sperber et al., 2019). However, advanced multivariate approaches (such as Lesymap’s sccan) are tailored to produce meaningful results from small sample datasets. It may be that the input of a whole-brain CBF map to the multivariate brain-behavior analysis allowed the model to converge because the entire network is represented in the data input, rather than a subset often defined by a lesion overlap mask.

Another limitation of this study is the MRI sequence parameters used to collect perfusion-weighted images. Our pCASL MRI sequence used a 2D EPI acquisition without background suppression and a single post-labeling delay. The current recommendations of the field (Alsop et al., 2015) are to use a 3D acquisition to remove the difference in blood arrival between slices and to apply background suppression to improve the computation of the difference signal by reducing the overlying tissue signal. We chose not to use background suppression because we are interested in quantifying the CBF of the lesion, but the T1 of lesioned tissue is not known and requires an additional MRI scan to quantify. Therefore, background suppression in ASL sequences must still be optimized for the chronic stroke brain, but will likely improve the relationship between lesion CBF and tissue damage quantified using TIGR. The lesion CBF of S10 likely suffered from an inadequate subtraction and caused the relationship between CBF and tissue damage to be positive, when all other participants had the expected negative relationship. Further, the intact WM CBF was consistently quantified at ∼30 mL/100 g/min in this cohort, which is higher than the literature WM CBF of ∼20 mL/100 g/min (Mokhber et al., 2021), possibly due to an incomplete tissue subtraction. The other limitation of the pCASL MRI sequence used in this study is the single post-labeling delay of 2,200 ms, which is relatively long, but will not provide additional information about blood arriving at a later time due to collateral flow (Lou et al., 2019). It may be ideal to collect two or more PLDs (perhaps up to 2,800 ms) to improve upon the CBF quantification after stroke or use the more advanced MR ASL fingerprinting (Su et al., 2017) to quantify multiple physiological parameters such as CBF and bolus arrival time using a single acquisition. Collecting MRS ASL fingerprinting data could also rule out arterial transit time artifacts that may occur in and around the lesion, which may be responsible for the “jump” in CBF midway through the tissue damage scores. The increased information from MR ASL fingerprinting within chronic stroke lesions could help improve the modeling of demographic and stroke-related factors, as well as brain-behavior relationships.

The removal of physiological noise from the perfusion-weighted signal was also not undertaken. We did not collect either cardiac or respiratory information during the pCASL MRI acquisition, as such data was not readily available using our setup. It has been shown that removing both cardiac and respiratory fluctuations during the ASL analysis can help improve the stability of the signals and remove spurious regional variations (Hassanpour et al., 2018). It may be that the slight negative CBF of highly damaged (i.e., cavitated) regions in some participants may be a result of physiological pulsatility causing a greater label signal compared to the control signal. Another means of removing physiological noise in ASL images is to use ICA-denoising approaches (Carone et al., 2019), but optimization and development of ICA denoising on stroke ASL data was beyond the scope of this report.

## Conclusion

In summary, we presented for the first time that CBF can be detected in chronic stroke lesions, demonstrating that this signal relates to tissue damage, with adequate fidelity to model with stroke and demographic-related factors and to relate to behavior.

## Data Availability

The raw data supporting the conclusion of this article will be made available by the authors, without undue reservation.
